# Structural characterization and electrochemical properties of Co_3_O_4 _anode materials synthesized by a hydrothermal method

**DOI:** 10.1186/1556-276X-7-73

**Published:** 2012-01-09

**Authors:** Chorong Shin, James Manuel, Dul-Sun Kim, Ho-Suk Ryu, Hyo-Jun Ahn, Jou-Hyeon Ahn

**Affiliations:** 1 Department of Chemical and Biological Engineering and Engineering Research Institute, Gyeongsang National University, 900, Gajwa-dong, Jinju, 660-701, Republic of Korea; 2 World Class University Center for Next Generation Battery, Gyeongsang National University, 900, Gajwa-dong, Jinju, 660-701, Republic of Korea

**Keywords:** cobalt oxide, hydrothermal method, anode material, lithium battery

## Abstract

Cobalt oxide [Co_3_O_4_] anode materials were synthesized by a simple hydrothermal process, and the reaction conditions were optimized to provide good electrochemical properties. The effect of various synthetic reaction and heat treatment conditions on the structure and electrochemical properties of Co_3_O_4 _powder was also studied. Physical characterizations of Co_3_O_4 _are investigated by X-ray diffraction, scanning electron microscopy, and Brunauer-Emmett-Teller [BET] method. The BET surface area decreased with values at 131.8 m^2^/g, 76.1 m^2^/g, and 55.2 m^2^/g with the increasing calcination temperature at 200°C, 300°C, and 400°C, respectively. The Co_3_O_4 _particle calcinated at 200°C for 3 h has a higher surface area and uniform particle size distribution which may result in better sites to accommodate Li^+ ^and electrical contact and to give a good electrochemical property. The cell composed of Super P as a carbon conductor shows better electrochemical properties than that composed of acetylene black. Among the samples prepared under different reaction conditions, Co_3_O_4 _prepared at 200°C for 10 h showed a better cycling performance than the other samples. It gave an initial discharge capacity of 1,330 mAh/g, decreased to 779 mAh/g after 10 cycles, and then showed a steady discharge capacity of 606 mAh/g after 60 cycles.

## Introduction

High performance batteries are receiving more attention nowadays, and many battery types are being developed for commercial use. Lithium ion batteries are known to be extremely safe and more reliable than rechargeable batteries with lithium metal because the charge-discharge reaction between a carbon negative electrode and a positive electrode consisting of metal oxide deals only with lithium ion. Research on the anode material for lithium ion battery has been focused on carbonaceous materials and alternative materials like tin oxide, cobalt oxide, etc. [1]. Carbonaceous materials have high stability but low volumetric capacity mainly due to their large initial irreversible capacity. In the last two decades, a number of various insertion compounds have been investigated with respect to their use as possible electrode materials in rechargeable lithium ion batteries.

Transition metal oxides consisting of layered structures or three-dimensional frameworks with tunnel systems accept remarkable amounts of lithium. Nanoscale or micron-sized transition metal oxides are promising alternative anode materials with excellent electrochemical performance for lithium ion batteries. Cobalto-cobaltic oxide belongs to a cubic closely packed structure of oxide ions and has attracted comparatively more attention due to the broad range of applications such as heterogeneous catalysts [2,3], anode materials in lithium ion rechargeable batteries [4], solid-state sensors [5], electrochromic devices [6], solar energy absorbers [7-9], magnetic materials [10], and ceramic pigments [11]. Cobalt oxide [Co_3_O_4_] is a good candidate as an anode material for lithium secondary batteries because of its good electrochemical capacity and high recharging rate [12]. Various synthetic routes have been developed for the preparation of Co_3_O_4 _such as thermal deposition [13], chemical spray pyrolysis [14], chemical vapor deposition [6], pulsed laser deposition [15], and traditional sol-gel method [16]. These methods need a relatively high temperature, and it is difficult to obtain nanocrystalline Co_3_O_4_. However, hydrothermal synthesis has emerged as an attractive and simple route for the preparation of such metal oxide nanoparticles. Jiang et al. reported the hydrothermal synthesis of Co_3_O_4 _by a two-step synthetic route using CoSO_4_·7H_2_O, ammonia, and H_2_O_2 _at 180°C [17].

In this work, Co_3_O_4 _powder was prepared by a hydrothermal process, and the reaction conditions were optimized to provide good electrochemical properties. The effect of various reaction conditions and heat treatment on the structure and electrochemical properties of Co_3_O_4 _powder was also studied. Cobalt nitrate hexahydrate (Co(NO_3_)_2_·6H_2_O) as the source of cobalt, hexamethylenetetramine [HMT] (C_6_H_12_N_4_) as a precipitator, and sodium citrate dihydrate (C_6_H_5_Na_3_O_7_·2H_2_O) as a template were used for the preparation of Co_3_O_4_.

## Experimental details

Co_3_O_4 _was synthesized by a simple hydrothermal reaction. In a typical procedure, cobalt nitrate hexahydrate, HMT, and sodium citrate dihydrate were added to 100 ml of deionized water with stirring. The mole ratio used for Co(NO_3_)_2_:HMT:C_6_H_5_Na_3_O_7 _was 6:3:2. After stirring for 10 min, the solution was kept in a Teflon-lined stainless steel autoclave and heated to temperatures in the range of 150°C to 250°C for different time durations (6 to 20 h) in a nitrogen atmosphere. The precipitate was collected by centrifugation and washed successively with distilled water and absolute alcohol, and dried at 75°C for 24 h. The precipitate obtained was heated at 200°C to 400°C for 3 h in air.

The characterization of prepared samples was done by X-ray powder diffraction [XRD] (D5005, BRUKER AXS GMBH, Karlsruhe, Germany using an X-ray diffractometer with Cu Kα radiation over the range of 10° to 90° 2*θ*), and surface morphology was studied using scanning electron microscopy [SEM] (JEOL 5600, JEOL Ltd, Akishima, Tokyo, Japan and TESCAN VEGAIILMU, Czechoslovakia). The specific surface area of the samples was measured using the Brunauer-Emmett-Teller [BET] procedure (ASAP 2010, Micromeritics Instrument Co., Norcross, GA, USA) from the N_2 _adsorption-desorption isotherms.

The anode was prepared by mixing Co_3_O_4 _powder, a carbon conductor (Super P carbon black or acetylene black [AB] (Alfa Aeser, Ward Hill, MA, USA)), and a poly(vinylidene fluoride) (PVdF, Sigma-Aldrich, St. Louis, MO, USA) binder in a 70:20:10 weight ratio. The ingredients were mixed in a high energy mixer mill for 1 h at room temperature using *N*-methylpyrrolidone as solvent to get a homogenous slurry which was cast on copper foil and dried at 80°C under vacuum for 12 h. The film was cut into circular disks of 1-cm^2 ^area and approximately 1.5-mg mass for use as anode. Electrochemical coin cells were assembled with lithium metal (300 μm in thickness; Cyprus-Foote Mineral Co., Kings Mountain, NC, USA) as cathode, Celgard^® ^2200 (Celgard LLC, Charlotte, NC, USA) as separator, 1 M LiPF_6 _in ethylene carbonate/diethyl carbonate (Samsung Cheil Industries, Gyeonggi-do, South Korea; 1:1, *v*/*v*) as electrolyte, and Co_3_O_4 _as anode. The cell assembly was performed under an argon atmosphere in a glove box (H_2_O < 10 ppm). The charge-discharge tests were carried out using an automatic galvanostatic charge-discharge unit, WBCS3000 battery cycler, between 0.001 and 2.5 V at a current density of 0.1 C.

## Results and discussions

**Figure 1:**
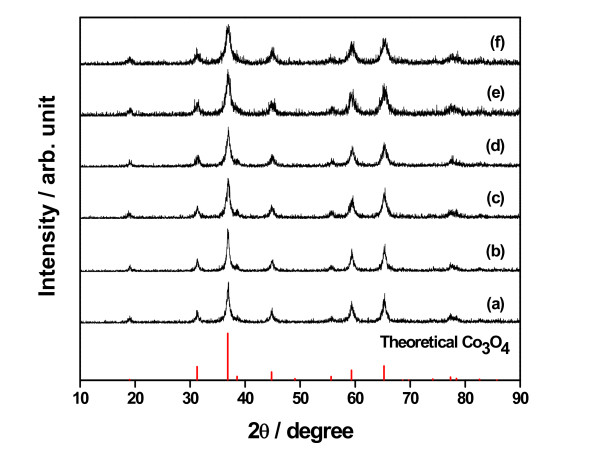
**XRD patterns of samples calcined at 200°C for 3 h prepared under different reaction conditions**. (**a**) 150°C for 20 h, (**b**) 180°C for 12 h, (**c**) 200°C for 6 h, (**d**) 200°C for 10 h, (**e**) 200°C for 12 h, and (**f**) 250°C for 6 h.

**Figure 2:**
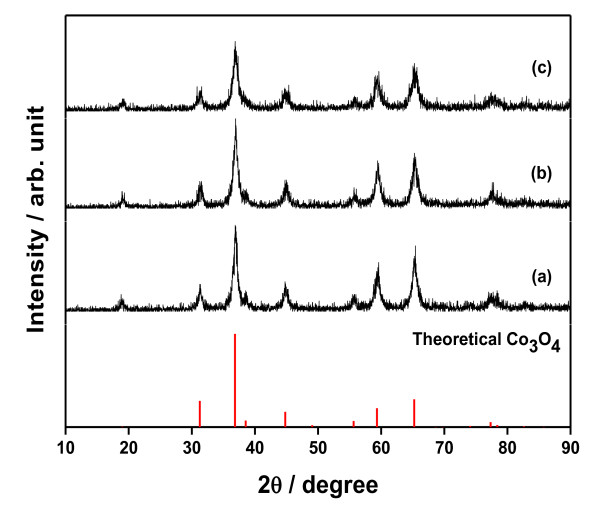
**XRD patterns of samples prepared at 200°C for 10 h under different calcination temperatures**. (**a**) 200°C for 3 h, (**b**) 300°C for 3 h, and (**c**) 400°C for 3 h.

The structural characterization of the prepared samples was done by using XRD. XRD patterns of the samples prepared under different reaction conditions and then calcined at the same condition of 200°C for 3 h are given in Figure 1. All the peaks shown in the figure correspond to the cubic spinel structure of Co_3_O_4_. The XRD patterns have main diffraction peaks at 36.9°, 65.2°, and 31.2° corresponding to (311), (440), and (220) reflections, respectively. No impurity peaks were observed, indicating the high purity of the final products. When the reaction condition of 150°C and 180°C was selected, at least 20 and 12 h were required to obtain a single phase with well-crystallized Co_3_O_4_, respectively. XRD patterns of the samples prepared at 150°C for 12 h and 180°C for 10 h indicated the presence of impurity, which is likely due to the insufficient reaction condition. All the samples prepared at 200°C for different time spans showed high purity peaks. For the sample prepared at 200°C, a 6-h time span was enough to get high purity peaks. Figure 2 shows XRD patterns of the samples prepared at 200°C for 10 h and calcined at different temperatures, 200°C, 300°C, and 400°C. XRD patterns of three samples are consistent and follow the same pattern as the theoretical one. There is no change in the chemical or crystalline structure of Co_3_O_4 _during heat treatment at different temperatures.

**Figure 3:**
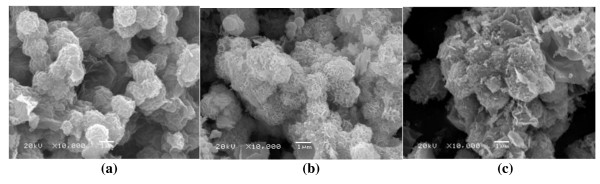
**SEM images of samples prepared at 200°C for 10 h and calcinated at different temperatures**. (**a**) 200°C for 3 h, (**b**) 300°C for 3 h, (**c**) 400°C for 3 h.

The surface morphology of the prepared products was examined by SEM. SEM images of Co_3_O_4 _materials prepared under different calcination temperatures were investigated. When the calcination temperature was increased, the surface of the particle became rough, and the particle cracked, as shown in Figure 3. The Co_3_O_4 _particles calcinated at 200°C for 3 h are of the microsphere type and have a uniform particle size distribution. The BET surface area decreased with values at 131.8 m^2^/g, 76.1 m^2^/g, and 55.2 m^2^/g with the increasing calcination temperature at 200°C, 300°C, and 400°C, respectively. The Co_3_O_4 _particles calcinated at 200°C for 3 h have a high surface area and uniform particle size distribution which may result in the good cycling property of the material.

Sodium citrate dihydrate is widely used to make microsphere type particles as a chelate complex and stabilizing agent in nanocolloidal chemistry, which is rendered by three carboxyl anions. These carboxyl anions can adsorb on the surface of metal ions and exert either hydrophobic or coulombic effects on metal ions and thereby act as a template to form and stabilize spherical structures [18]. With the effect of sodium citrate dihydrate, the thickness of layers gradually increases in a concave shape resulting in microsphere formation [19,20]. The ability of HMT to capture protons might accelerate the formation of crystalline subunits during the gradual hydrolysis of HMT, thereby formation of more amorphous spheres might occur [21].

**Figure 4:**
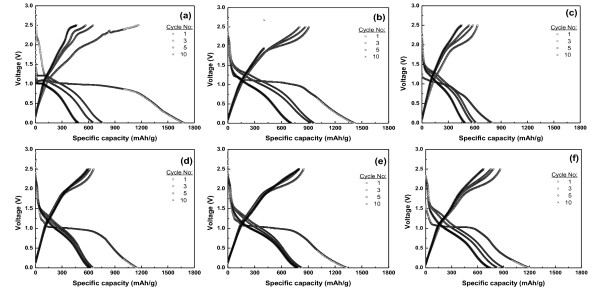
**Charge-discharge curves of Co_3_O_4 _electrodes using AB and Super P as carbon conductors**. At 0.1 C-rate, between 0.001 and 2.5 V, Co_3_O_4 _electrodes were prepared at (**a**) 150°C for 20 h, (**b**) 200°C for 6 h, (c) 250°C for 8 h with AB; and (**d**) 150°C for 20 h, (**e**) 200°C for 6 h, (**f**) 250°C for 8 h with Super P.

The charge-discharge properties of the cells with Co_3_O_4 _electrode were evaluated between 0.001 and 2.5 V at a current density of 0.1 C-rate. Figure 4 shows the charge-discharge curves of Co_3_O_4 _electrodes using AB and Super P as conductors. The overall initial electrochemical processes can be expressed as follows:

$$\text{C}{\text{o}}_{\text{3}}{\text{O}}_{\text{4}}+\text{ 8L}{\text{i}}^{+}+\text{ 8}e^{-}\;\overset{}{\underset{}{\to }}\text{ 3Co }+\text{ 4L}{\text{i}}_{\text{2}}\text{O}$$.

The theoretical capacity is 890 mAh/g. The sudden drop in specific capacity of the cell after the first cycle is due to the irreversible reaction of Co_3_O_4_. It could be seen that the potential drops rapidly, followed by a plateau at about 1.0 V, and a gradual decrease to the cutoff voltage of 0.001 V for the lithium reaction during the initial discharge process as shown in Figure 4. However, the plateau disappeared during the second cycle. The initial capacities of the cell using AB as the carbon conductor are ordered as follows: the samples prepared at 150°C for 20 h (1,680 mAh/g), 200°C for 10 h (1,420 mAh/g), and 250°C for 8 h (798 mAh/g). All values of the initial capacities of Co_3_O_4 _electrodes using Super P and AB carbon conductors were significantly higher than the theoretical capacity. The capacities of the Co_3_O_4 _electrode using the Super P carbon conductor decreased rapidly, and the plateau disappeared during the second cycle just like the Co_3_O_4 _electrode using AB. In comparison, the cell using AB as conductor delivered a higher initial discharge capacity than the cell using Super P, but the capacity drop is higher in the former.

**Figure 5:**
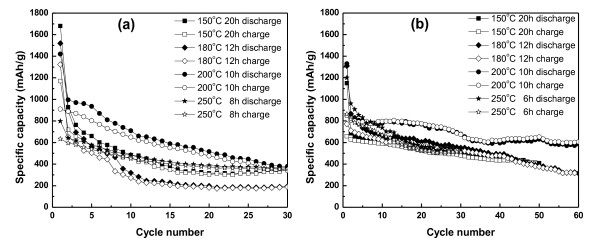
**Cycling behavior of the cells based on Co_3_O_4 _electrodes prepared at different reaction conditions**. Reaction conditions with (**a**) AB as carbon source; (**b**) Super P carbon black as carbon source (voltage range 0.001 to 2.5 V versus Li/Li^+^; 0.1 C-rate).

**Figure 6:**
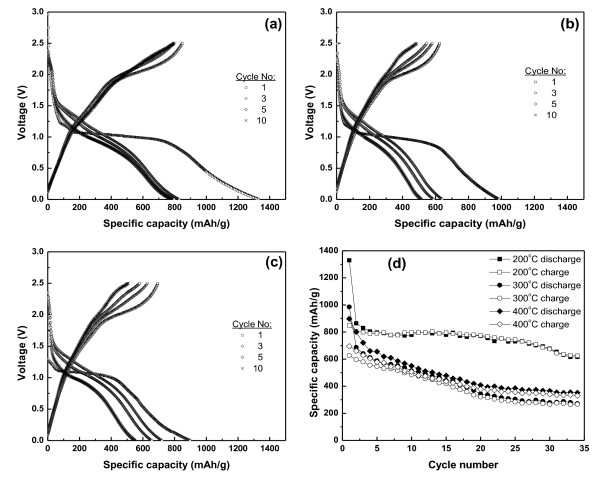
**Cell charge-discharge curves based on Co_3_O_4 _prepared with different heat treatment conditions**. Prepared at 200°C for 10 h, the Co_3_O_4 _were heat treated at (**a**) 200°C for 3 h; (**b**) 300°C for 3 h; (**c**) 400°C for 3 h; (**d**) cycling behavior of Co_3_O_4 _electrodes (voltage range 0.001 to 2.5 V versus Li/Li^+^; 0.1 C-rate).

Figure 5 shows the overall cycle property of Co_3_O_4 _electrodes. The figure shows the comparison of the cycle performance of the cells using Co_3_O_4 _prepared at different conditions with AB (Figure 5a) and Super P (Figure 5b) as conductors. From Figure 5a, the cycle property of Co_3_O_4 _prepared at 200°C for 10 h showed a better cycling performance with a high specific capacity than that of the other samples. The cell showed an initial discharge capacity of 1,420 mAh/g and suddenly dropped to 935 mAh/g after 5 cycles; after that, it delivered a good cycle performance compared with the other samples. In the case of the cells using Super P carbon black as the carbon conductor, the cycle property of the sample prepared at 200°C for 10 h has good electrochemical properties such as initial capacity, low capacity fading, and long-life stable cycle performance. It reached 606 mAh/g after 60 cycles which is 68% of the theoretical capacity. This can be attributed to the uniform particle size and to the rough and porous structure of the material which provide a large surface area and thereby availability of the large number of active sites for lithium ion intercalation. In Figure 6a, b, it could be seen that the cell with Super P carbon black has better electrochemical and cycle properties than that with AB. It is clear that Co_3_O_4 _prepared at 200°C for 10 h showed a better cyclic performance with the Super P carbon conductor than with other samples.

Figure 6 showed the comparison of cycle performance of the cells based on Co_3_O_4 _prepared at 200°C for 10 h and then calcined at different temperatures, 200°C, 300°C, and 400°C, for 3 h. The cells made of Co_3_O_4 _calcined at 200°C, 300°C, and 400°C delivered initial discharge capacities of 1,330, 985, and 898 mAh/g which dropped to 677, 279, and 365 mAh/g after 30 cycles, respectively. The sample calcined at 200°C for 3 h showed a better electrochemical property than the other samples which is due to the occurrence of cracks in the samples during high temperature treatment. The sample prepared at 200°C for 3 h has a uniform particle size and high surface area which can provide better sites to accommodate Li^+ ^and electrical contact, and give a good electrochemical property.

## Conclusions

Co_3_O_4 _particles were synthesized by a simple hydrothermal method at various reaction and heat treatment conditions from cobalt nitrate, hexamethylenetetramine, and sodium citrate dehydrate. Depending on the synthetic conditions, different morphologies were obtained, which noticeably affected the electrochemical properties. The cells with Super P as a carbon conductor showed better electrochemical properties than the cells with AB. The discharge capacity decreased with the increasing cycle number but still remained relatively high. Among the samples prepared under different reaction conditions, Co_3_O_4 _prepared at 200°C for 10 h showed a better cycling performance than the other samples.

## Competing interests

The authors declare that they have no competing interests.

## Authors' contributions

CS and DSK are the primary authors. They both conceived the study, carried out the experiments, characterization, acquisition of data, analysis and interpretation of data, drafting of the manuscript, and revisions. JM and HJA participated in language modification and also in interpretation of data. HSR participated in the analysis and interpretation of data. JHA is the principal investigator. All authors read and approved the final manuscript.
